# Effects of Nitrate- and Betalain-Rich Beetroot Juice Ingestion on Vascular Function and Metabolic Syndrome Risk Factors in Healthy Adults: A Pilot Study

**DOI:** 10.3390/metabo16080579

**Published:** 2026-08-17

**Authors:** Cameron Haswell, Kay Rutherfurd-Markwick, Roger Hurst, Marie Wong, Hajar Mazahery, Ajmol Ali, Rachel Page

**Affiliations:** 1 School of Health Sciences, Massey University, Auckland 0745, New Zealand; c.haswell@massey.ac.nz (C.H.); k.j.rutherfurd@massey.ac.nz (K.R.-M.); 2 Ag Emission Centre, New Zealand Institute for Bioeconomy Science Limited (AgResearch Group), Grasslands Research Centre, Palmerston North 4410, New Zealand; hurstroger1@gmail.com; 3 School of Food Technology and Natural Sciences, Massey University, Auckland 0745, New Zealand; m.wong@massey.ac.nz; 4 Curtin School of Population Health, Curtin University, Perth, WA 6102, Australia; hajar.mazahery@curtin.edu.au; 5 Institute for Physical Activity and Nutrition (IPAN), School of Exercise and Nutrition Sciences, Deakin University, Geelong, VIC 3220, Australia; 6 School of Sport, Exercise and Nutrition, Massey University, Auckland 0745, New Zealand; a.ali@massey.ac.nz; 7 School of Health Sciences, Massey University, Wellington 6140, New Zealand

**Keywords:** type 2 diabetes, cardiovascular health, dietary intervention, systemic vascular resistance, blood pressure, MetS

## Abstract

**Background/Objectives:** Health challenges associated with metabolic syndrome (MetS) have stimulated investigations into the effectiveness of nutraceutical interventions, including beetroot juice (BRJ), in mitigating MetS risk factors. **Methods:** This repeated-measures crossover trial (*n* = 16 healthy participants) investigates the effects of daily ingestion of two different BRJs [United Kingdom beetroot juice (UKBR), 1280 mg nitrate, 147 mg betalains; New Zealand beetroot juice (NZBR), 1120 mg nitrate, 506 mg betalains] and placebo (PL) on plasma nitrate and nitrite levels, MetS biomarkers and systemic vascular resistance (SVR). Participants consumed the juices for 6 days, with measures taken on day 7 fasted (pre-dose) and 2 h and 5 h post ingestion (acute). **Results:** On day 7, pre-dose plasma nitrate levels were significantly elevated for UKBR and NZBR compared to PL (297.2, 177.1 and 64.2 µmol/L, respectively). On day 7, UKBR achieved significantly higher plasma nitrate levels 2 h and 5 h post consumption than NZBR (2 h: 750.3 vs. 577.7 µmol/L; 5 h: 703.4 vs. 510.1 µmol/L) and PL at all timepoints. Plasma nitrite was elevated pre-dose for UKBR only and for both juices relative to PL at 2 h and 5 h. On day 7, pre-dose systolic blood pressure (SBP) was significantly lower for NZBR than PL (−4.96 mmHg) and 2 h post-ingestion for both UKBR (−5.63 mmHg) and NZBR (−4.98 mmHg) compared with PL. Systemic vascular resistance was 11.6% lower after 6 days of consumption of NZBR relative to PL (ratio 0.884, 95% CI 0.803 to 0.974). **Conclusions:** Supplementation with BRJ reduced SBP relative to placebo, and the betalain-rich NZBR also reduced SVR, whereas the higher-nitrate UKBR did not. This suggests that betalains and nitrates may play an important role in these improvements. The Australia New Zealand Clinical Trials Registry ID for this study is: ACTRN12622000714785.

## 1. Introduction

Metabolic syndrome (MetS) and type 2 diabetes mellitus (T2DM) represent a growing global health issue, posing significant challenges to public health systems, economies, and population well-being. Metabolic syndrome is a term describing a cluster of metabolic abnormalities associated with increased risk of T2DM, cardiovascular disease (CVD) and development of coronary heart disease (CHD) [1,2]. Metabolic syndrome comprises five components, including hypertension, central obesity, low levels of high-density lipoprotein cholesterol (HDL-C), hypertriglyceridemia and impaired fasting glucose [3]. Each component contributes to, or characterises, the development of insulin resistance, prediabetes and subsequent T2DM. An individual is diagnosed with MetS if they exhibit three of the five risk factor components. Like T2DM, MetS is associated with physical inactivity and central obesity, and an individual with MetS has a fivefold relative risk of developing T2DM [4].

Approximately one-quarter of the global adult population has MetS [5], posing a significant risk for increased global T2DM rates. With T2DM prevalence increasing worldwide, its global economic and social burden is also growing; in the United States, for example, currently 25% of the national healthcare cost is accredited to T2DM management and treatment [6], whilst the projected annual cost of T2DM in New Zealand (NZ) will rise from NZ$2.1 billion to NZ$3.5 billion (63% increase) between 2022 and 2045 [7]. Much of this cost is associated with increased prevalence of T2DM in younger people, with lifetime healthcare costs more than tenfold for someone diagnosed with T2DM at 25 years compared to someone diagnosed at 75 years [7].

The significant prevalence, healthcare costs and social burden of MetS and T2DM underscores the urgency to find effective interventions to prevent the onset of MetS risk factors and subsequent development of T2DM. Dietary interventions may provide a low-cost, accessible strategy to mitigate these risk factors and beetroot juice (BRJ) supplementation has attracted interest due to its rich composition of bioactives such as nitrate and betalains [8,9,10].

Dietary nitrate, found in high levels in BRJ, reduces to nitric oxide (NO), a regulator of blood vessel function [11,12], lipid peroxidation [13] and glucose metabolism [14]. Previous studies show that BRJ consumption increases plasma nitrate and nitrite levels 2 h post-ingestion and they remain elevated after 6 h [15,16], with NO metabolites [17] and total nitroso species [18] also elevated. Beetroot juice is also a source of the polyphenolic pigments betalains, and polyphenols further enhance the reduction of nitrate to NO. Betanin, a sub-class of betalains, may also inhibit both α-amylase and α-glucosidase, decreasing the rate of carbohydrate breakdown and glucose absorption into the blood [19].

Previous studies investigating the impact of beetroot (BR) supplementation on improving metabolic syndrome biomarkers have yielded positive results. Several studies have shown BRJ consumption leads to clinically significant reductions in blood pressure following both acute [16,20,21] and longer-term [22,23,24,25,26] supplementation, with most chronic supplementation studies showing reductions in both systolic blood pressure (SBP) and diastolic blood pressure (DBP) between 5 and 10 mmHg. As dietary nitrate is reduced into nitrite and subsequently to NO, NO bioavailability increases. Nitric oxide activates guanylyl cyclases, which catalyse the conversion of guanosine triphosphate into cyclic 3’, 5′-guanosine monophosphate in the vascular smooth muscle cells, reducing intracellular Ca^2+^ levels, which decreases smooth muscle contraction, leading to vasodilation and decreasing blood pressure [27]. Despite this, evidence of the effect of BRJ intake on systemic vascular resistance (SVR), which, unlike SBP, is regulated primarily by the microvasculature [28], is limited. Systemic vascular resistance, defined as the resistance offered by the systemic vasculature to the flow of blood from the left ventricle into the arterial circulation, reflects the degree of arteriolar vasoconstriction or vasodilation and is a key determinant of mean arterial pressure. Systemic vascular resistance may be a better indicator of early hypertension risk in healthy people [29], as microvascular endothelial dysfunction tends to precede macrovascular endothelial dysfunction [30], making SVR a useful measure in studies predicting the effect of interventions on future hypertension risk in healthy people.

Previous studies have shown that BRJ and dietary nitrate supplementation may improve glucose metabolism. Gheibi and colleagues [31] showed increased glucose transporter-4 (GLUT4) protein expression in T2DM rats following dietary nitrate supplementation, due to increased GLUT4 mRNA expression. Nitric oxide also contributes to vasodilation, causing relaxation of large blood vessels, which increases glucose delivery to peripheral tissues [32,33]. Holy and colleagues [32] showed a reduction in triglycerides (TG), total cholesterol (TC), and low-density lipoprotein cholesterol (LDL-C) levels in healthy subjects following an acute dose of BRJ (250 mL, nitrate levels not specified). The betalain pigment betanin has been shown to inhibit lipid peroxidation [34], leading to reduced reactive oxygen species (ROS) levels; however, other work has shown that lipid profiles can be improved from dietary nitrate consumption in the absence of betalains [35].

Based on available evidence, consumption of BRJ may have the potential to ameliorate MetS risk factors and reduce the overall risk of MetS and onset of T2DM [36]. The exact mechanisms behind these observations are not fully established, and previous studies [16,20,21,22,23,24,25,26,31] have failed to establish the relative contributions of betalains and dietary nitrate on the observed effects.

The levels of nitrate and betalains in BRJ are known to vary based on the cultivar utilised [10,37], growth conditions (sowing date [37], length of growth period [8,10], and nitrogen supply [37]) as well as production methods [38], resulting in considerable variability in content between different BRJ brands [8] as well as different batches of the same product [8]. Health benefits from consuming BRJ may therefore differ based on the relative composition of the bioactive ingredients (nitrate and betalains) in the products.

This pilot study aimed to investigate the effects of intake of two different BRJs with different betalain and nitrate content on plasma nitrate and nitrite levels and MetS risk factors (BP, lipid and blood glucose levels) following 6 days of daily BRJ consumption and then examine acute changes in plasma nitrate and nitrite, blood pressure, SV and SVR following ingestion on day 7. This pilot study was performed in healthy adults to explore the potential of the BRJ bioactives (betalains and dietary nitrates) eliciting beneficial changes in blood glucose and lipid levels and vascular function measures before undertaking larger studies aimed at investigating their effect in at-risk populations such as people with MetS.

## 2. Materials and Methods

### 2.1. Ethics and Regulatory Approvals

Ethics approval was obtained from the Massey University Human Ethics Committee (Southern A, Application 21/36—granted on 4 October 2021) and was prospectively registered at the Australia New Zealand Clinical Trials Registry (Trial ID: ACTRN12622000714785). All participants were required to give written informed consent prior to participation in the study. No formal patient or public involvement was undertaken in the design, conduct, analysis, or reporting of this trial. The study protocol, participant-facing materials, and procedures were reviewed and approved by the relevant institutional ethics committee, which included lay/public representation.

### 2.2. Study Population

Healthy participants were recruited from Auckland, New Zealand, using a combination of poster advertising, social media outreach and word of mouth. Inclusion criteria for the study were: (i) aged 18–55 years (and premenopausal if female), (ii) BMI of 18–30 kg/m^2^, and (iii) able to cycle at moderate intensity for 120 min. Participants were excluded from the study if they were: (i) diagnosed with diabetes or any other chronic illness (e.g., infectious blood-borne diseases and cardiovascular diseases) which might affect the study results or cause difficulty in exercising, (ii) smokers, (iii) had a history of diabetes, cardiovascular disease or hypertension, and (iv) allergic to BR intake.

#### Sample Size

Data for this study was collected as part of a larger project aimed at establishing the efficacy of beetroot juice supplementation to improve cycling time trial performance. A priori sample size calculations were performed based on plasma nitrate data reported by Lansley and colleagues [39]. Assuming a standard deviation of 4.5, a minimum sample size of 12 participants was estimated to achieve 80% power at α = 0.05. To account for a 20% potential participant dropout, at least 15 participants need to be recruited.

### 2.3. Study Design

The study was conducted as a repeated-measures, randomised, crossover trial with three testing arms: New Zealand beetroot juice (NZBR), United Kingdom beetroot juice (UKBR), and placebo (PL). On receiving written consent, the participant was invited to attend a familiarisation session (FAM) at the Massey University Innovation Complex, Albany, Auckland, Aotearoa, New Zealand, where the study was conducted. This FAM session enabled the researchers to guide the participants through the study requirements. At the FAM session, baseline parameters (age, height, body mass, vascular function, SV, SVR, SBP, DBP, fasting blood glucose and lipid profile) were measured to allow for comparison of these parameters after 6 days of supplementation with the intervention drink. These measures were obtained from finger-prick blood samples for fasting capillary blood glucose (HemoCue Glucose 201+ System, Hemocue, Ängelholm, Sweden) and lipid profile, with TC, HDL-C and TG determined by enzymatic assay and LDL-C by difference (Cobas^®^ b 101 system, Roche Diagnostics, Basel, Switzerland). Blood pressure was measured using a sphygmomanometer (Omron Smart+ Blood Pressure Cuff, Omron Corporation, Kyoto, Japan), following a 5 min period of seated rest with the mean of 2 measures used for analysis. If the initial 2 readings had a discrepancy of >5 mm Hg, a third reading was taken and the reading most different to the other two discarded. Vascular function (stroke volume (SV) and SVR) was determined by continuous-wave Doppler ultrasound cardiac output monitoring (USCOM; USCOM 1A, USCOM Ltd., Sydney, Australia) at the suprasternal notch, following the same 5 min rest period. Probe position was optimised using real-time measurement feedback and a single trace captured at the point of maximal signal quality. All measurements were taken by the same trained operator. Body mass (Inbody 270 Body Composition Analyser, Inbody Co., Ltd., Seoul, Republic of Korea), height (via stadiometer) and body mass index (BMI—calculated from body mass and height) were also measured. There was no indication of individuals with high muscle mass as measured by BIA.

**Figure 1:**
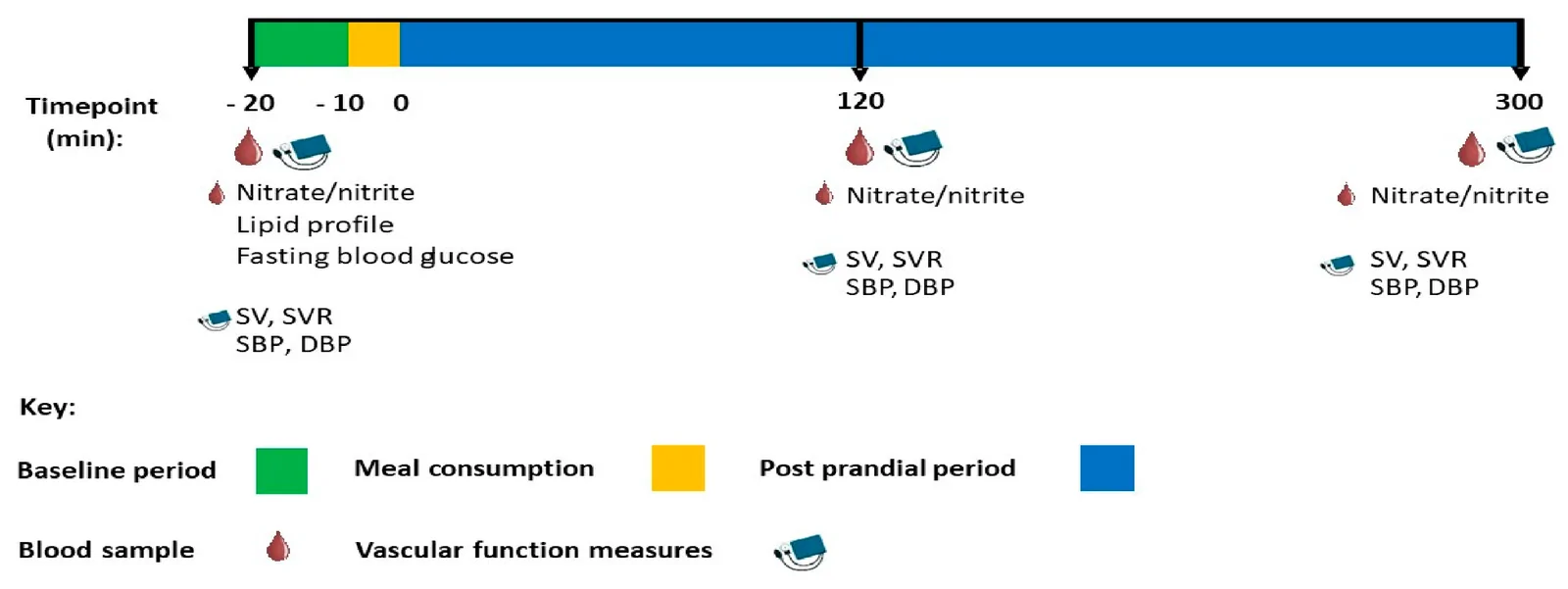
Study timeline for day 7, following 6 days of supplementation with each treatment. Fasted pre-dose (−20 min) samples were taken prior to the consumption of the test meal, and participants were asked to consume the meal and juice within 10 min. Time point 0 signals the end of test meal consumption. Vascular function measures: SV, stroke volume; SVR, systemic vascular resistance; SBP, systolic blood pressure; and DBP, diastolic blood pressure.

At the end of the FAM session, the participants were provided with their first randomised intervention drink (labelled A, B or C) in an opaque container and told to drink the intervention drink daily for 6 days and then attend the laboratory fasted on day 7. The timeline of events on day 7 is shown in Figure 1. A washout period of 7 days occurred before the participant started consuming the next randomised intervention drink.

Participants were asked to maintain their habitual diet and lifestyle behaviours throughout the supplementation period, other than consumption of the provided intervention. To minimise acute dietary and behavioural influences on outcome measures, participants were asked to avoid alcohol, caffeine, and strenuous exercise for 24 h before each study visit. Participants were also asked to avoid nitrate-rich foods, polyphenol-rich foods, and nutritional supplements for 7 days before each study visit. These instructions were applied consistently across all intervention conditions.

Study trial visits 1, 2 and 3 took place after participants had completed 6 days’ supplementation with one of the three drinks. The study timeline for testing on day 7 is shown in Figure 1. Participants arrived at the laboratory on day 7, after observing at least a 10 h fast, and provided finger prick blood samples for measures of fasting blood glucose and lipid profile.

Measures of SVR, SV, SBP, DBP, and venous samples for plasma nitrate and plasma nitrite were taken prior to (pre-dose, time 0 h) and at 2 h and 5 h post-ingestion of a breakfast meal consisting of either peanut butter on white toast (energy = 1138 kJ, protein = 14.8 g, carbohydrates = 30.0 g, fats = 10.0 g) or muesli with dairy milk and yoghurt (energy = 1351 kJ, protein = 15.0 g, carbohydrates = 30.0 g, fats = 10.2 g), alongside the intervention drink, all of which participants were asked to consume within a 10 min period. Between the 2 h and 5 h timepoints, participants completed a 2 h cycling protocol followed by a 10 km cycling time trial as part of the larger trial’s testing procedures. For males, each trial was followed by a minimum 7-day washout period before the initiation of supplementation with another drink. Females were required to attend their study trial visits during the early follicular phase of their menstrual cycle, or days 3–7 of their contraceptive placebo, resulting in a longer washout period (minimum 28 days). There was no significant carryover of either nitrate (*p* = 0.521) or nitrite (*p* = 0.282) between treatments, indicating the washout period was sufficient. There were no significant changes to the trial design, eligibility criteria, interventions, outcomes, or prespecified analyses after trial commencement.

#### Randomisation

Randomisation was blinded to the researchers (who enrolled the participants and performed the data collection and analysis) and participant. Treatment order for the participants was randomised across six possible sequences, balanced for sex. Randomisation was performed by an external researcher. The intervention drinks were labelled A, B and C and were presented in an opaque container. Even though the volume of one of the drinks differed and the taste and colour may have differed between the three intervention drinks, neither the participants nor the researchers knew which drink (UKBR, NZBR and placebo) was allocated to A, B or C. The placebo was designed to colour match the NZBR. Blinding success was not formally assessed.

### 2.4. Nitrate and Betalain Content of the Study Juices

The nitrate concentrations of the juices (three replicates analysed in duplicate) were measured by high-performance liquid chromatography (HPLC) using methods adapted from Chou and colleagues [40].

Betalain levels in the beverages were measured by spectrophotometric analysis using the methods and equations from Stintzing et al. [41] and Slatnar et al. [42]. Beetroot juice sourced from the UK (Beet-it, James White Drinks, Ipswich, UK) contained 1280 mg of dietary nitrate per dose (2 × 70 mL) and 147 mg betalains per dose, whilst the NZBR (made in-house) contained 1120 mg of nitrate and 506 mg of betalains per dose (2 × 100 mL), and placebo (made in-house) contained 103.2 mg nitrate per dose (2 × 100 mL). The betalain and nitrate content of the BRJs are shown in Table 1.

Participants consumed two bottles (1 dose) of either UKBR, NZBR or PL juice per day for 6 days prior to each of the study trial visits. Participants were allowed to consume the drinks at any time within the 24 h period of each day; however, they were asked to drink the whole content of at least one bottle at any given ingestion time. Participants were asked to return their empty bottles.

### 2.5. Blood Sampling and Analysis

Blood samples for plasma nitrate and nitrite were collected via venepuncture at the antecubital fossa into lithium heparin vacutainers (6 mL) pre-dose (post-6 days, time 0 h), 2 h and 5 h post-meal/beverage consumption. Samples for plasma nitrate and nitrite were collected at trial visits only. Capillary samples by finger prick were used for glucose and lipid analysis at familiarisation and trial visits. Vacutainers were centrifuged at 1500× *g* for 10 min; then, 500 µL of separated plasma was aliquoted into microcentrifuge tubes and stored at −80 °C for later analysis. Nitrate and nitrite plasma samples were then analysed in duplicate using HPLC methods. Plasma nitrite was analysed in accordance with previous methods adapted from Li and colleagues [43], whilst plasma nitrate was analysed by taking 500 µL of filtered plasma prepared during nitrite analysis and analysed according to the method described by Chou et al. [40]. Quality control parameters were determined as described in Chou et al. [40] and Li et al. [43], with all results being the same or slightly improved.

### 2.6. Harms

Harms were defined as any unfavourable or unintended sign, symptom, illness, or adverse experience occurring during the trial, whether or not it was considered related to the intervention. Adverse events were assessed non-systematically through participant self-report. Participants were encouraged to report any adverse symptoms or concerns throughout the study. Reported events were recorded and reviewed by the research team, including assessment of seriousness and likely relatedness to the intervention. No adverse events were reported.

### 2.7. Statistical Analysis

All outcomes were analysed using linear mixed-effects models fitted by restricted maximum likelihood in IBM SPSS Statistics version 31 (IBM Corp., Armonk, NY, USA), with participants treated as the subject of repeated measurements. Fixed effects were treatment, timepoint and their interaction. Treatment sequence and visit order were each examined and were not significant for any outcome (sequence, *p* > 0.80 in all indices; period, *p* > 0.10 in all indices). Therefore, neither sequence nor period were retained in final models.

For each outcome, compound symmetry, heterogenous compound symmetry and unstructured covariance structures were compared, and the structure with the lowest corrected Akaike information criterion (AICC) was retained. AICC was preferred to AIC due to the small sample size of the study. Compound symmetry was selected for all haemodynamic responses and blood chemistry outcomes and heterogenous compound symmetry for plasma nitrate and nitrite.

Systemic vascular resistance was analysed after logarithmic transformation due to right-skewed residuals resulting from it being a ratio measure. Model comparisons for this outcome are reported as ratios of geometric means with 95% confidence intervals; estimated means are reported as back-transformed absolute values. Plasma nitrate and nitrite were modelled at pre-dose, 2 h and 5 h. Blood pressure, stroke volume and SVR were modelled at pre-dose and 2 h only, the 5 h timepoint being excluded for these outcomes because it followed the exercise component of the trial. Analyses included all randomised participants and used all available observations; mixed models retain participants with incomplete data under a missing-at-random assumption, and no data were imputed.

Pairwise comparisons were made between estimated marginal means and are reported as mean differences with 95% confidence intervals. As this is an exploratory pilot study in which the sample size was determined for plasma nitrate analysis, pairwise comparisons were not adjusted for multiple comparisons. A *p*-value below 0.05 was considered statistically significant. No interim analyses were planned or conducted. No formal stopping guidelines were specified due to the short duration and low-risk nature of the intervention.

## 3. Results

### 3.1. Participants

**Figure 2:**
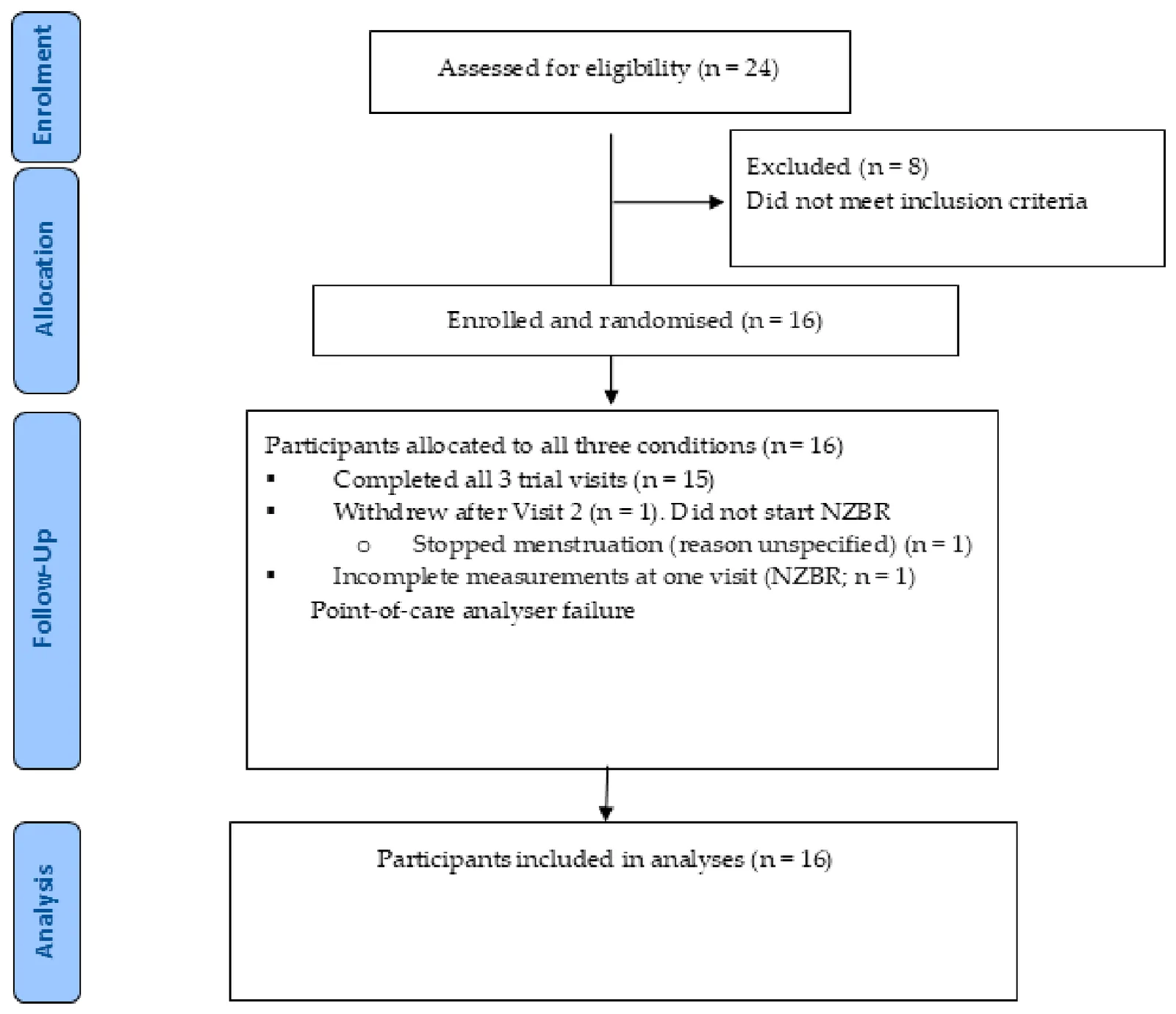
Study CONSORT flow diagram. Twenty-four participants were screened, sixteen were randomised, and fifteen completed all three trial visits. All 16 randomised participants were included in the analyses. M, male. F, female.

A total of 24 participants were screened, with 16 participants meeting all inclusion criteria (Figure 2). A total of 16 participants, 9 male (M) and 7 female (F), were randomised and took part in the study between July 2022 and May 2023, with 1 female participant withdrawing from the study before completion of their third visit (Figure 2); this participant did not withdraw consent, and her data from the two conditions she completed are included. In one male participant, a point-of-care analyser failure at one trial visit meant that capillary blood glucose and lipid measurements, and the accompanying haemodynamic measurements, were not obtained for that condition. There were no harms or adverse events reported by participants.

Baseline characteristics of the 16 participants measured at the FAM session who completed the whole study are shown in Table 2.

### 3.2. Plasma Nitrate and Nitrite Analysis

**Figure 3:**
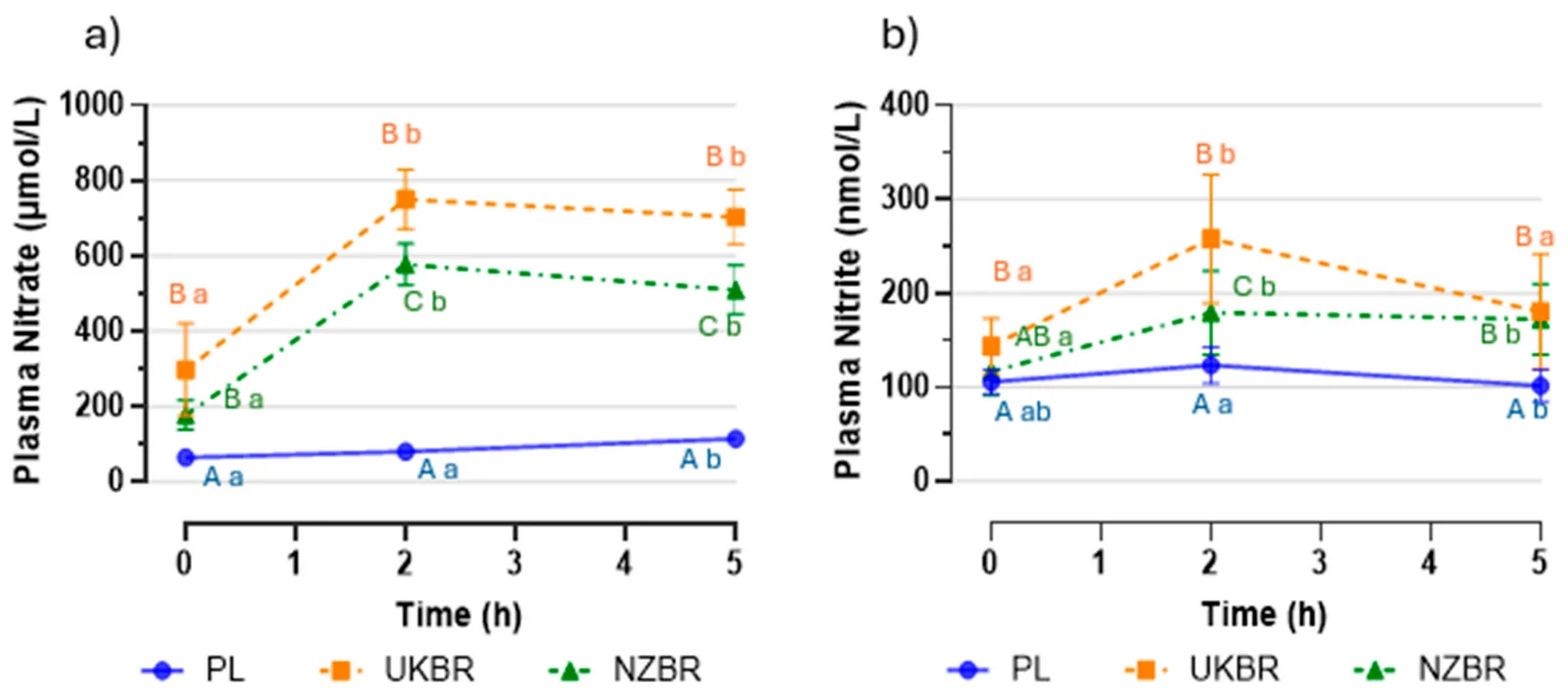
Plasma nitrate (**a**) and nitrite (**b**) concentrations after 6 days of supplementation with either placebo (PL), UK beetroot juice (UKBR) or NZ beetroot juice (NZBR). Timepoint at 0 h represents the pre-dose sample taken on day 7. Values are estimated marginal means from linear mixed-effects models (*n* = 16), with error bars representing 95% confidence intervals. Different uppercase letters denote a significant difference between conditions at that timepoint; different lowercase letters denote a significant difference between timepoints within a condition (*p* < 0.05). Conditions or timepoints sharing a letter did not differ significantly, and letters are assigned independently at each timepoint.

Plasma nitrate and nitrite were analysed for all 16 participants (9 M, 7 F), with 1 female participant not completing the NZBR condition. Plasma nitrate analyses showed a significant treatment × time interaction (F = 54.4, *p* < 0.001, Figure 3a). Consumption of both UKBR and NZBR resulted in higher plasma nitrate concentrations than PL pre-dose (0 h), 2 h and 5 h post-ingestion (all *p* ≤ 0.001). Moreover, the UKBR resulted in higher plasma nitrate concentrations than the NZBR treatment at 2 h (UKBR = 750.3 µmol/L, NZBR = 577.7 µmol/L; difference 172.6 µmol/L, 95% CI 87.7 to 257.5, *p* < 0.001) and 5 h (UKBR = 703.4 µmol/L, NZBR = 510.1 µmol/L; difference 193.3 µmol/L, 95% CI 108.9 to 277.7, *p* < 0.001) but not pre-dose (UKBR = 297.2 µmol/L, NZBR = 177.1 µmol/L; difference 120.1 µmol/L, 95% CI −0.1 to 240.4, *p* = 0.050).

Plasma nitrite analyses showed a significant treatment x time interaction (F = 4.96, *p* = 0.001, Figure 3b). The UKBR treatment yielded higher plasma nitrite concentrations than PL pre-dose (difference 38.2 nmol/L, 95% CI 11.5 to 64.9, *p* = 0.007), but no differences were observed between nitrite concentrations following the NZBR and PL treatments pre-dose (difference 11.2 nmol/L, 95% CI −11.9 to 34.2, *p* = 0.324). At 2 h, the UKBR treatment led to higher plasma nitrite concentrations than both the NZBR treatment (UKBR = 257.8 nmol/L, NZBR = 179.4 nmol/L; difference 78.4 nmol/L, 95% CI 15.7 to 141.1, *p* = 0.016) and the PL treatment (PL = 123.4 nmol/L; difference 134.4 nmol/L, 95% CI 71.7 to 197.2, *p* < 0.001), whilst nitrite concentrations at 2 h following the NZBR treatment were also higher than the PL treatment (difference 56.0 nmol/L, 95% CI 15.5 to 96.6, *p* = 0.009). At 5 h post-ingestion, no differences in plasma nitrite concentrations were observed between the participants consuming the UKBR and NZBR treatments (difference 8.1 nmol/L, 95% CI −48.3 to 64.5, *p* = 0.769); however, both the UKBR (difference 79.0 nmol/L, 95% CI 22.5 to 135.5, *p* = 0.009) and NZBR (difference 70.9 nmol/L, 95% CI 36.9 to 104.9, *p* < 0.001) treatments resulted in higher plasma nitrite concentrations than the PL treatment.

### 3.3. Blood Pressure and Systemic Vascular Resistance

**Figure 4:**
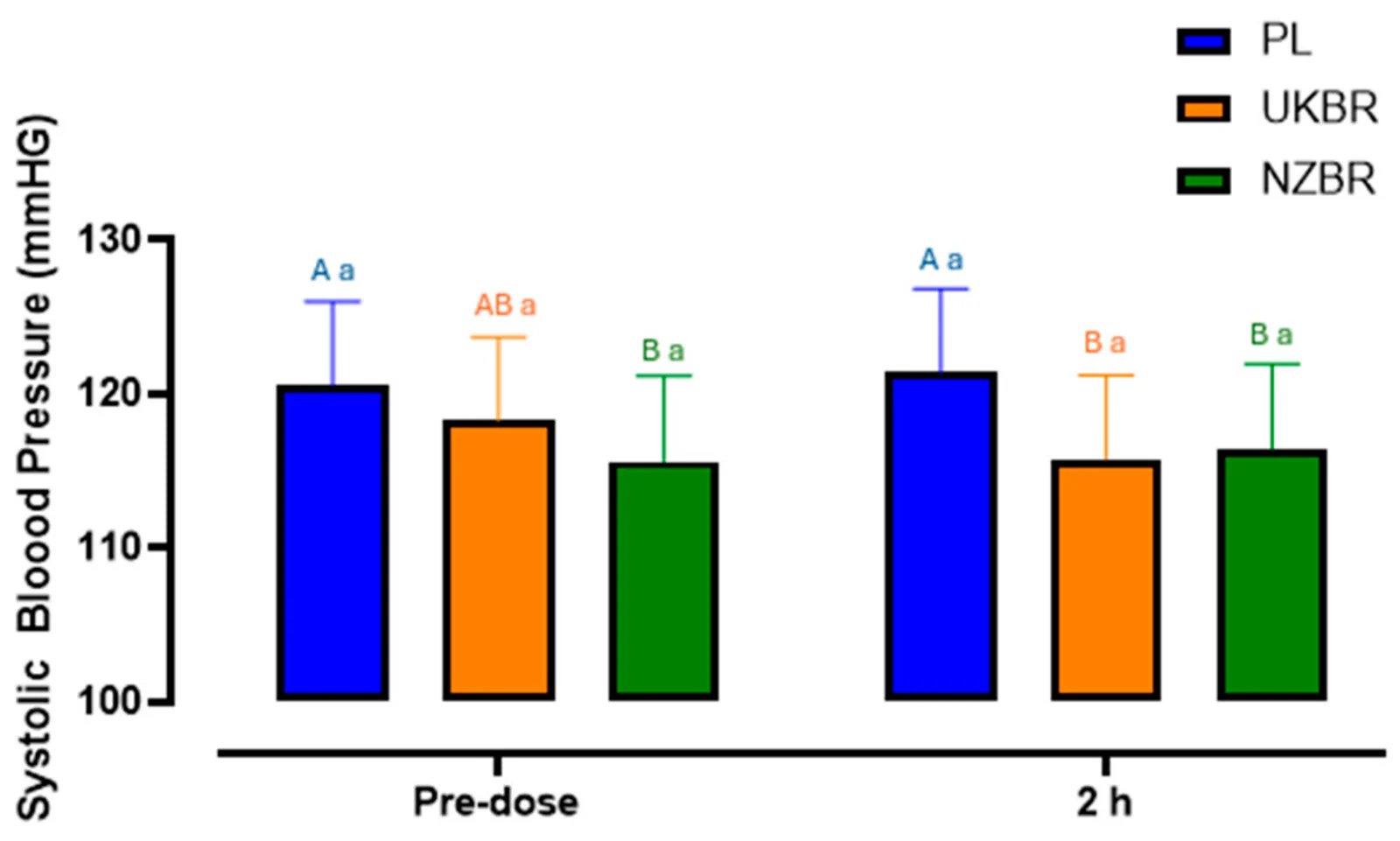
Systolic blood pressure after 6 days of supplementation with either placebo (PL), UK beetroot juice (UKBR) or NZ beetroot juice (NZBR). Pre-dose represents the measurement taken on day 7 before the final dose and 2 h the post-meal timepoint on day 7. Values are estimated marginal means from linear mixed-effects models (*n* = 16) with error bars representing 95% confidence intervals. Different uppercase letters denote a significant difference between conditions at that timepoint; different lowercase letters denote a significant difference between timepoints within a condition (*p* < 0.05). Letters are assigned independently at each timepoint.

All 16 participants were included in blood pressure and vascular function analyses. For SBP, there was an effect of treatment (F = 5.85, *p* = 0.004) but not of time (F = 0.06, *p* = 0.811) or of the treatment x time interaction (F = 0.79, *p* = 0.456). Consumption of the NZBR treatment resulted in lower SBP after 6 days of supplementation (pre-dose) than PL (−4.96 mmHg, 95% CI −9.35 to −0.56, *p* = 0.028). Further, 2 h post-meal and ingestion of BRJ, SBP was lower in the UKBR and NZBR conditions than PL (PL = 121.4 mmHg; UKBR = 115.8 mmHg, difference −5.63 mmHg, 95% CI −9.84 to −1.41, *p* = 0.010; NZBR = 116.4 mmHg, difference −4.98 mmHg, 95% CI −9.38 to −0.58, *p* = 0.027; Figure 4).

**Figure 5:**
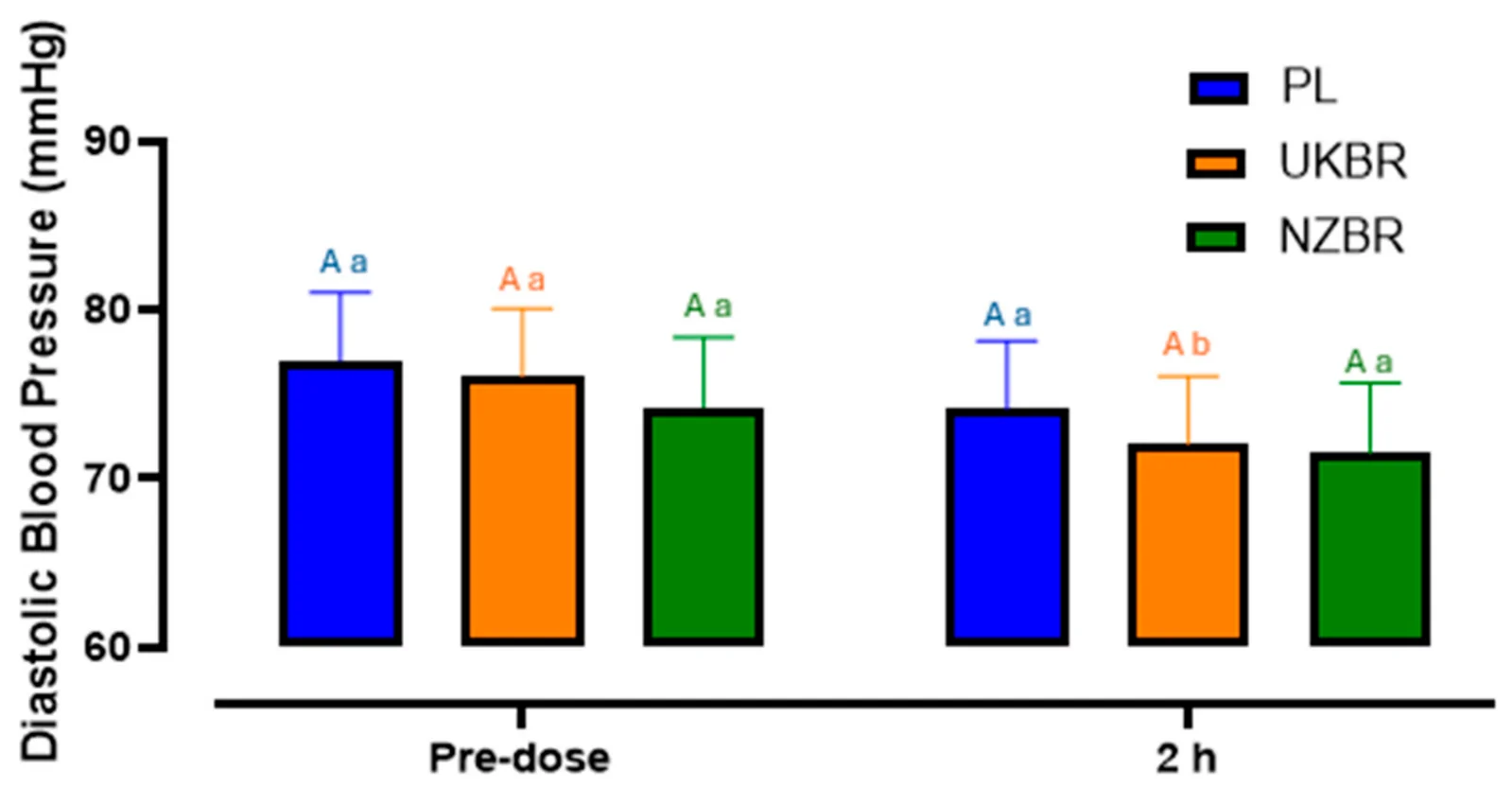
Diastolic blood pressure after 6 days of supplementation with either placebo (PL), UK beetroot juice (UKBR) or NZ beetroot juice (NZBR). Pre-dose represents the measurement taken on day 7 before the final dose and 2 h the post-meal timepoint on day 7. Values are estimated marginal means from linear mixed-effects models (*n* = 16), with error bars representing 95% confidence intervals. Different uppercase letters denote a significant difference between conditions at that timepoint; different lowercase letters denote a significant difference between timepoints within a condition (*p* < 0.05). Letters are assigned independently at each timepoint.

For DBP, there was no effect of treatment (F = 2.28, *p* = 0.110) or of the treatment × time interaction (F = 0.16, *p* = 0.850), but there was a significant effect of time (F = 10.02, *p* = 0.002). DBP was lower 2 h post-meal than pre-dose across all three conditions, consistent with a postprandial response rather than an effect of supplementation. There were no differences in DBP between treatments after 6 days of supplementation or 2 h post-meal (all *p* ≥ 0.088, Figure 5).

There was an effect of time (F = 15.61, *p* < 0.001), with SV increasing from pre-dose (0 h) to 2 h post-meal across all three conditions. There was no effect of treatment (F = 2.23, *p* = 0.115) or treatment x time (F = 0.69, *p* = 0.506) for SV. No significant differences were observed between participant SV at FAM and after 6 days of supplementation in any condition.

**Figure 6:**
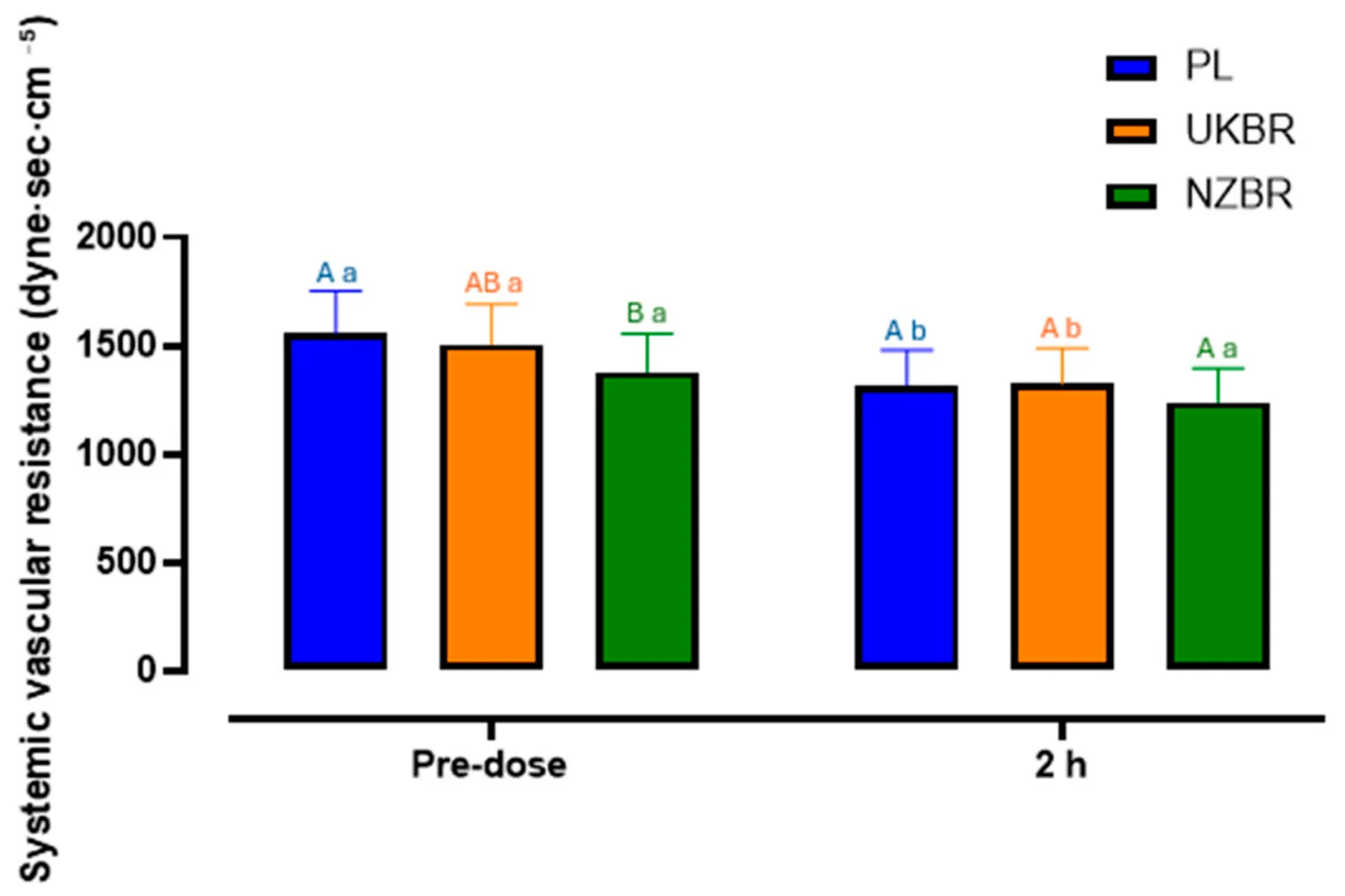
Systemic vascular resistance after 6 days of supplementation with either placebo (PL), UK beetroot juice (UKBR) or NZ beetroot juice (NZBR). Pre-dose represents the measurement taken on day 7 before the final dose and 2 h the post-meal timepoint on day 7. SVR was analysed following natural logarithm transformation; means are back-transformed and confidence intervals are therefore asymmetric. Values are estimated marginal means from linear mixed-effects models (*n* = 15, one participant excluded owing to an outlying familiarisation value), with error bars representing 95% confidence intervals. Different uppercase letters denote a significant difference between conditions at that timepoint; different lowercase letters denote a significant difference between timepoints within a condition (*p* < 0.05). Letters are assigned independently at each timepoint.

SVR was analysed following natural logarithm transformation and is reported as ratios of geometric means; one participant was excluded because of an outlying familiarisation value of 3309 dyne·s·cm^−5^ [44]. There was an effect of treatment (F = 3.96, *p* = 0.024) and of time (F = 23.99, *p* < 0.001) but not of the treatment × time interaction (F = 0.45, *p* = 0.639). After 6 days of supplementation, SVR was 11.6% lower following the NZBR treatment than PL (ratio 0.884, 95% CI 0.803 to 0.974, *p* = 0.013), whereas the UKBR treatment did not differ from PL (ratio 1.035, 95% CI 0.944 to 1.136, *p* = 0.446). SVR was 8.4% lower following NZBR than UKBR, but this comparison did not reach statistical significance (ratio 0.916, 95% CI 0.832 to 1.009, *p* = 0.074, Figure 6). SVR decreased from pre-dose to 2 h post-meal in the PL and UKBR conditions, with a similar but non-significant reduction following NZBR, in which SVR was already lower pre-dose. No differences between treatments were observed at 2 h (all *p* ≥ 0.197).

### 3.4. Blood Glucose and Lipid Levels

Comparisons of blood glucose and lipid parameters are presented in Table 3. All 16 participants contributed to these analyses. There were no differences in participants’ fasting blood glucose, total cholesterol, HDL-C, triglyceride or LDL-C concentrations between FAM and after 6 days of supplementation with any of the intervention drinks (all *p* > 0.18).

## 4. Discussion

The aim of this study was to assess and compare the effects of consuming two different BRJ beverages on metabolic syndrome risk factors after 6 days of daily juice consumption. We also aimed to compare the impact of acute ingestion of two different BRJ beverages on measures of vascular function, blood pressure, and plasma nitrate and nitrite levels. By investigating BRJ containing different concentrations of nitrates and betalains, we aimed to gain a better understanding of the contributions of these bioactive compounds to any observed changes in the parameters measured. The results from this study were consistent with previous findings that BRJ supplementation reduces blood pressure compared to placebo [16,20,21,45] and showed that SVR was reduced following consumption of the betalain-rich NZBR but not the UKBR.

As expected, plasma nitrate concentrations were elevated following 6 days of ingestion of UKBR and NZBR compared to PL. Simultaneously, plasma nitrite concentrations were elevated in the UKBR compared to PL after 6 days of ingestion. Previous studies have also shown an accumulation of nitrite in the blood following short-term dietary nitrate supplementation (4–6 days with 372 mg nitrate/day) [46,47]. McDonagh and colleagues used a similar protocol, providing 2 × 70 mL shots of BRJ containing ~400 mg of nitrate and measured increases in plasma nitrate and nitrite acutely at 2 h and 5 h post-ingestion, as well as pre-dose on day 7 after 6 days of supplementation [48]. A placebo containing 103.2 mg nitrate/day was used in the present study reflecting the real-life dietary intake of nitrate in humans [49]. In the current study, the plasma nitrate and nitrite concentrations for the PL group are similar or less than those reported pre-supplementation in the study by Stanaway et al. [16]. Therefore, the use of this low-nitrate placebo is unlikely to have had an effect on the differences between the placebo and the BR interventions as both conditions resulted in significant changes in plasma nitrate and nitrite following acute ingestion and the levels of plasma nitrate and nitrite remained relatively low in the placebo group over time.

The present study showed that plasma nitrite concentrations were elevated above placebo following 6 consecutive days of supplementation (pre-dose day 7) with UKBR (1280 mg nitrate/day) but not NZBR (1120 mg/day) and acutely following ingestion of both juices. After 6 days of supplementation with the two juices the plasma nitrate (*p* = 0.050) and nitrite (*p* = 0.067) pre-dose values did not differ from each other. Whereas following the acute dose UKBR produced higher concentrations of both. This suggests that differences in circulating nitrate and nitrite between these two nitrate doses are more readily detected acutely than after a loading period.

Even after 6 days of daily consumption, an acute dose significantly increased both plasma nitrate and nitrite concentrations following both UKBR and NZBR consumption compared to the placebo. The UKBR resulted in higher plasma nitrate levels than NZBR at both the 2 h and 5 h timepoints (although exercise may have affected the 5 h timepoint values), as well as higher nitrite levels at the 2 h timepoint. Given the UKBR treatment had a higher nitrate dose than the NZBR, this suggests a dose–response relationship between the amount of ingested dietary nitrate and subsequent circulating plasma nitrate levels, a precursor for NO production which contributes to the maintenance of vascular homeostasis by vascular endothelial cells and vascular smooth muscle cells [50].

In this study, SBP did not change between pre-dose and 2 h post-ingestion in any condition. DBP fell from pre-dose to 2 h across all three conditions (*p* = 0.002) with no difference between treatments (*p* = 0.110), consistent with a postprandial response rather than an effect of supplementation. Beetroot juice supplementation has been shown to acutely decrease both SBP and DBP [16,20,21], and a comparison of meta-analyses shows that those with only a hypertensive adult cohort show greater reductions in SBP (−4.96 mmHg) [51] than meta-analyses that included both hypertensive and healthy participants (−3.55 mmHg) [52]. The present study used participants who had normotensive SBP, but they still showed reductions in SBP of ~5 mmHg between groups consuming NZBR juice and placebo pre-dose on day 7 (after 6 days of supplementation) and 2 h post-supplementation. This result is encouraging as a 5 mmHg reduction in SBP is clinically significant, as it reduces the risk of cardiovascular events by 10%, with similar patterns reported for DBP [53].

Supplementation on day 7 did not cause an acute reduction in SBP compared to pre-dose in the UKBR or NZBR interventions. This may suggest that a ‘saturation point’ of nitrite utilisation in normotensive healthy young individuals is reached after the 6-day loading period, meaning further reductions in SBP are not possible. Whilst this may be the case in our study, previous research in older adults has shown continued improvements after 28 days of supplementation [16]. Further, plasma nitrite concentrations after 6 days were significantly higher following UKBR than PL, whereas NZBR did not differ from PL; yet it was the NZBR condition in which SBP was significantly lower than PL. It appears the reduction in SBP following short-term BRJ ingestion may not be solely due to the blood-pressure-lowering effects of dietary nitrate but other compounds, such as betalains, which were present at higher concentrations in the NZBR, may be contributing to the observed reductions in SBP following the NZBR intervention. Shepherd and colleagues [45] reported a reduction in BP following ingestion of nitrate-depleted beetroot juice (NDBR), highlighting that the lowering of blood pressure observed in previous studies may not be caused entirely by nitrate intake alone. There is evidence that plasma concentrations of the anti-inflammatory cytokine interleukin-10 (IL-10), which increases endothelial NO expression [54] and inhibits angiotensin-II-induced vascular dysfunction [55], increase following ingestion of both BRJ and NDBR [45] and also that betalain-rich dyes augment IL-10 production in mouse models [56,57]. Although speculative, this pathway may explain why SBP was reduced relative to placebo following consumption of the betalain-rich NZBR but not following UKBR. This warrants further investigation. The nitrate-independent potential for betalains to reduce blood pressure has been corroborated by a study [58] comparing the effectiveness of *Opuntia stricta* (prickly) pear cactus and red beetroot. *Opuntia stricta* is known to be comparable to BR in betalain content [59] but ~20 times lower in dietary nitrate [60]. Individuals with atherosclerotic cardiovascular disease were supplemented with either *Opuntia stricta* or red BR for two weeks, with both treatments showing improvements in SBP compared to placebo. However, greater reductions were observed in the red BR juice condition [58].

Significant reductions in SVR were observed after 6 days of supplementation (pre-dose) with NZBR compared with PL (11.6% lower, ratio 0.884, 95% CI 0.803 to 0.974, *p* = 0.013), whereas UKBR did not differ from PL (*p* = 0.446). SVR was 8.4% lower following NZBR supplementation than following UKBR supplementation, but this direct comparison did not reach statistical significance (ratio 0.916, 95% CI 0.832 to 1.009, *p* = 0.074). The effect observed with the NZBR is notable because SVR is primarily controlled by the microvasculature, where endothelial dysfunction tends to precede the development of hypertension [29]. The NZBR contained lower levels of nitrate but higher levels of betalains than the UKBR, and betalains have been shown to have strong antioxidant and anti-inflammatory effects [61], which may improve microvascular endothelial function. However, this is a preliminary study and further research is required.

Systemic vascular resistance was also reduced 2 h postprandially in the PL and UKBR conditions, and showed a similar but non-significant reduction following NZBR, in which SVR was already lower pre-dose. Previous studies have shown splanchnic blood flow is increased following meal consumption to secure essential nutrients and oxygen to the intestines for digestion and absorption of digested substances to the liver [62,63]. This is caused by vasodilation of blood vessels supplying the stomach, intestines, pancreas and liver, which is primarily mediated by increases in NO signalling [64]. Due to this vasodilation of microvascular blood flow, SVR is reduced throughout the whole body, meaning any acute changes to SVR caused by ingesting the BRJ may have been masked by the effect of concurrent meal consumption.

Despite prior studies suggesting that acute consumption of BRJ can improve lipid profiles [65,66,67,68] and glucose metabolism [67,69,70,71], in this study, no significant changes were observed in fasting blood glucose, triglycerides, or cholesterol levels after 6 days of supplementation. There are several possible explanations for these findings. Firstly, the low number of participants and relatively short duration of the intervention (6 days) may have been insufficient to produce measurable changes in lipid metabolism or glucose homeostasis, which typically require more prolonged dietary interventions ranging from 4 to 12 weeks [72,73,74,75]. Secondly, the participants in this study were generally healthy and normolipidemic, so their baseline lipid and glucose levels were already within a normal range, potentially limiting the capacity for further improvement. HDL-C concentrations were nominally lower at all three trial visits than at FAM but by a near-identical margin in the placebo and both beetroot conditions (PL and UKBR did not differ *p* = 0.997). Future research should explore supplementation in populations at risk of hypercholesterolaemia or MetS over periods of 4 weeks or more to investigate the potential of BRJ consumption to improve glucose and lipid metabolism.

This preliminary study suggests that the different bioactive components in BRJ, nitrates and betalains, may produce beneficial health effects. While dietary nitrate clearly plays a role in acute vasodilation and blood pressure reduction, our findings that SVR was reduced in the NZBR juice group (but not in the lower betalain-containing UKBR juice) suggest that betalains may contribute to more sustained improvements in vascular function, particularly in the microvasculature. SVR was reduced relative to placebo following NZBR consumption but not following UKBR consumption, despite NZBR containing 160 mg less nitrate and 359 mg more betalains per daily dose (Table 1). The direct comparison between the two juices did not reach statistical significance, so this observation cannot establish an independent effect of betalains. However, it does suggest that betalains may have bioactivity complementing that of dietary nitrate, possibly by enhancing NO bioavailability [54]. This warrants investigation in a trial using nitrate-matched beverages differing only in betalain content.

Whilst this study provided insight into the effects of BRJ on plasma nitrate concentrations and haemodynamics, several limitations in the study design may have limited our understanding of the effects of BRJ supplementation on glucose control and lipid parameters. Firstly, the small sample size of this study limits the generalisability and statistical power of the study. This means that, even though we did not see any significant effects in the blood glucose and lipid levels, these non-significant results cannot be considered as conclusive evidence for lack of effect. Secondly, our study used a healthy, normotensive population capable of 2 h of continuous physical activity. The use of healthy, moderately trained participants likely limited the ability of the study to observe significant changes in compounds such as lipids and glucose as the body normally tightly controls the levels of these compounds within a very narrow range. Thirdly, the duration of the intervention (6 days) may have been insufficient for detecting more substantial changes in glucose metabolism and lipid profiles. Previous studies often involved longer intervention periods from 4 to 12 weeks [72,73,74,75] or used a population already at risk of MetS, such as those with hypercholesterolaemia or T2DM [76,77].

A further limitation is that the two juices used in this study were not nitrate-matched (UKBR: 1280 mg vs. NZBR: 1120 mg per daily dose). This modest difference in nitrate content clearly influenced circulating nitrate/nitrite kinetics as reflected in the higher plasma nitrate/nitrite in the UKBR condition. There was a larger difference in the amount of betalains (UKBR: 147 mg vs. NZBR: 506 mg per daily dose) between the two BRJ. These differences in nitrate and betalain concentrations between the two juices complicates attribution of vascular effects to betalains versus nitrate. Consequently, the possibility that between-arm differences in SBP and SVR reflect interactions between dose-dependent NO bioavailability and betalain content rather than betalains alone cannot be dismissed.

This study was part of a larger project exploring the effect of different types of beetroot juices on exercise performance. Therefore, with the addition of 2 h of exercise within the protocol, the 5 h nitrate/nitrite measurements need to be treated with caution. Further, as the sample size was determined by plasma nitrate, all outcomes other than plasma nitrate and nitrite should be interpreted as exploratory, and pairwise comparisons were not adjusted for multiple testing.

To establish in greater depth the contribution of betalains to the improvements in vascular function seen in this study and other studies of its type, studies using NDBR, dietary nitrate without betalains and BRJ with both bioactives should be employed. Establishing the relative contributions of betalains and nitrates to mitigation of different MetS risk factors may help inform intervention strategies in the future.

Future studies should utilise a larger sample size, focus on populations at risk of MetS development or who already present with MetS risk factors and should equate nitrate dose and manipulate betalain content independently. This would allow gender differences to be investigated as well as providing more conclusive evidence of effects on specific risk factor markers. The importance of dietary nitrates vs. betalains and whether there is an additive and/or synergistic effect in eliciting health benefits could also be ascertained. Studies using a longer supplementation period should also be carried out and could include a wider range of biomarkers such as insulin, HOMA-IR, IL-6, IL-10, oxidative stress markers and endothelial function. This would provide more information related to the mechanisms of action of the bioactives in BRJ and how these may translate to health benefits.

## 5. Conclusions

This pilot study, carried out in healthy participants, showed that 6 days of supplementation with beetroot juice reduced systolic blood pressure relative to placebo and that the betalain-rich NZBR also reduced systemic vascular resistance, whereas the higher-nitrate UKBR did not. Plasma nitrate and nitrite increased more at the higher nitrate dose (UKBR), but this did not translate into a correspondingly greater improvement in vascular function. The direct comparison of change in SVR between the two juices did not reach statistical significance, therefore the contribution of betalains remains to be established. This preliminary data indicates the effect of betalains on SVR warrants further investigation. Future research should determine whether similar effects are observed in at-risk populations such as those with MetS and should establish the mechanisms underlying the observed effects.

## Figures and Tables

**Table 1:** Betalain and nitrate content of the study juices.

	UKBR		NZBR	
Daily Dose	2 × 70 mL		2 × 100 mL	
Compound	Relative proportion (%)	Total dose (mg)	Relative proportion (%)	Total dose (mg)
Betanin	27.9	41.0	42.5	215.2
Isobetanin	32.4	47.7	29.9	151.1
Neobetanin	36.4	53.5	26.1	132.0
Unknown	3.3	4.8	1.5	7.7
Total Betalains	100	147.0	100	506.0
Nitrate		1280		1120

Individual betalains are presented as a proportion of total betalain content and as absolute amounts per dose (2 bottles). Nitrate is presented as mg per dose (2 bottles). UKBR, United Kingdom beetroot juice; NZBR, New Zealand beetroot juice.

**Table 2:** Baseline characteristics of study participants stratified by sex.

Variable	Male (M)	Female (F)
*n*	9	7
Age (y)	28 ± 12	29 ± 13.7
Height (cm)	179.6 ± 3.2	166.9 ± 5.4
Body mass (kg)	80.1 ± 11.3	63.8 ± 9.5
BMI (kg/m^2^)	24.8 ± 3.5	22.8 ± 2.5
Vascular function		
SV (mL/beat)	79.6 ± 16.5	78.3 ± 10.1
SVR (dyne·sec·cm^−5^) ‡	1589 ± 355	1473 ± 352
Blood pressure		
SBP (mmHg)	128.6 ± 11.1	113.6 ± 10.7
DBP (mmHg)	77.2 ± 9.1	72.0 ± 81.3.5

Data are presented as mean ± standard deviation. BMI, body mass index; SV, stroke volume; SVR, systemic vascular resistance; SBP, systolic blood pressure; DBP, diastolic blood pressure. ‡ SVR for males calculated excluding one participant with an outlier baseline value of 3309 dyne·sec·cm^−5^ (*n* = 8).

**Table 3:** Fasting blood glucose and lipid profiles at familiarisation (FAM) and pre-dose on day 7, after 6 days of supplementation with either placebo (PL), UK beetroot juice (UKBR) or NZ beetroot juice (NZBR).

Blood Parameter	FAM	PL (Day 7)	*p*-Value	UKBR (Day 7)	*p*-Value	NZBR (Day 7)	*p*-Value
Fasting blood glucose (mmol/L)	4.94 (4.62, 5.25)	5.34 (5.04, 5.63)	0.064	5.14 (4.84, 5.45)	0.693	5.17 (4.85, 5.48)	0.297
Triglycerides (mmol/L)	1.40 (0.98, 1.81)	1.56 (1.16, 1.97)	0.517	1.21 (0.78, 1.64)	0.488	1.39 (0.96, 1.82)	0.976
Total cholesterol (mmol/L)	4.34 (3.88, 4.79)	4.16 (3.71, 4.61)	0.268	4.05 (3.59, 4.51)	0.092	4.32 (3.86, 4.78)	0.917
HDL-C (mmol/L)	1.62 (1.47, 1.77)	1.51 (1.36, 1.66)	0.052	1.51 (1.36, 1.66)	0.063	1.54 (1.39, 1.69)	0.176
LDL-C (mmol/L)	2.06 (1.63, 2.50)	1.94 (1.51, 2.36)	0.478	2.13 (1.69, 2.58)	0.726	2.14 (1.70, 2.58)	0.695

Values are estimated marginal means (95% confidence interval) from linear mixed-effects models, *n* = 16 for all parameters except NZBR, where *n* = 14. *p*-values are for comparison with FAM. FAM, familiarisation. HDL-C, high-density lipoprotein cholesterol. LDL-C, low-density lipoprotein cholesterol.

## Data Availability

The data presented in this study are available upon request from the corresponding author.

## Abbreviations

The following abbreviations are used in this manuscript:

MetS	Metabolic syndrome
T2DM	Type 2 diabetes mellitus
BR	Beetroot
BRJ	Beetroot juice
UKBR	Beetroot Juice sourced from the United Kingdom
NZBR	Beetroot Juice sourced from New Zealand
PL	Placebo beverage
SBP	Systolic blood pressure
DBP	Diastolic blood pressure
SVR	Systemic vascular resistance
CVD	Cardiovascular disease
CHD	Coronary heart disease
HDL-C	High-density lipoprotein cholesterol
NO	Nitric oxide
TG	Triglycerides
TC	Total cholesterol
LDL-C	Low-density lipoprotein cholesterol
SV	Stroke volume
BMI	Body mass index
HPLC	High Performance Liquid Chromatography
FAM	Familiarisation
TNF-α	Tumour necrosis factor-alpha
ROS	Reactive oxygen species
NDBR	Nitrate-depleted beetroot juice
